# Argumentation in anonymous online discussions about decriminalising cannabis use

**DOI:** 10.1177/14550725211027383

**Published:** 2021-08-10

**Authors:** Lasse Hämäläinen, Emmi Lahti

**Affiliations:** 7840 University of Tampere, Tampere, Finland; 3835 University of Helsinki, Helsinki, Finland

**Keywords:** argumentation, cannabis, discourse analysis, drug policy, rhetoric

## Abstract

**Aims:**

In October 2019, a citizens’ initiative to decriminalise cannabis use started a large debate about drug policy in Finland. This study examines online discussions about the initiative to supplement the current knowledge about citizens’ drug opinions. The focus is especially on argumentation techniques that are used to support or object to the decriminalisation.

**Design:**

Methodologically, the study is based on discourse studies, new rhetoric, and argumentation analysis. The data of 1,092 messages were collected from a popular Finnish anonymous discussion forum Ylilauta.

**Results:**

Online discussions about the legal status of cannabis are highly polarised. Decriminalisation is often both supported and resisted in a strong and affective manner, and even hate speech is not rare in the data. Statements made by both discussion parties often lack any argumentation or are based on fallacies, especially ad hominem arguments. Some discussants refer to scientific studies and expert statements, even though such references are usually inaccurate. Cannabis is compared to alcohol more often than to other illegal drugs.

**Conclusions:**

The emotional responses and inadequate argumentation might be partially explained by the general nature of online discussions and the culture of the investigated website, but also by the powerful stigma related to illegal drugs and insufficient knowledge on the subject. A future objective is to create a societal atmosphere where the complex question of the legal status of cannabis could be discussed more neutrally and rationally.

Growing, manufacturing, selling, and using cannabis products as well as certain other psychoactive substances has been illegal throughout most of the world for the past half century. During the previous few decades, however, this policy has been increasingly criticised and questioned, and some countries have started to reform their drug policies (see, e.g., Abalo, 2019). In Finland, the actions taken to renew the drug policy have been moderate (Hakkarainen et al., 2007), even though several experts and authorities have expressed the need for such reform (Humaania päihdepolitiikkaa ry., 2019).

In October 2019, however, an official citizens’ initiative to decriminalise cannabis use (Kansalaisaloite.fi, 2019) collected more than 50,000 signatures and made it to the Finnish Parliament for deliberation (for information on the citizens’ initiative system in Finland, see Kansalaisaloite.fi, 2021). The deliberation process had not been finished by April 2021, but in the news media it was deemed unlikely that the initiative would be accepted. Nevertheless, the initiative gave rise to a large societal debate about the current Finnish drug policy. Not only was the initiative discussed in the traditional media, but also on the internet and social media by ordinary citizens.

For scholars, the debate provided an opportunity to update and expand knowledge regarding Finnish citizens’ opinions and attitudes towards drugs, and on the other hand, to examine the discursive construction of the debate. This study investigates how the cannabis debate is discursively constructed in an anonymous online forum and what kind of argumentation strategies are used. Moreover, it is discussed what this can tell us about the opinions and attitudes towards cannabis.

Thus far, scholarly knowledge about citizens’ drug opinions both in Finland and in several other countries has been largely based on population surveys. In Finland, probably the most important of these surveys has been the Finnish Drug Survey, carried out every four years by the Finnish Institute for Health and Welfare (THL). Its results have been presented and discussed extensively in various research reports (e.g., Hakkarainen, 1996; Hakkarainen et al., 2015; Karjalainen et al., 2017; Karjalainen et al., 2020), also with a special focus placed on drug attitudes (Hakkarainen & Metso, 2004) and cannabis (Hakkarainen & Karjalainen, 2017). According to the latest survey in 2018, 24% of the adult population in Finland have used cannabis at least once in their life, 42% think that cannabis use should not be punished, and 72% accept medical cannabis (Karjalainen et al., 2020). However, as the survey forms used are strictly structured and include few open fields, the image of citizens’ drug opinions drawn by the surveys is limited, especially from a qualitative standpoint (see also Hakkarainen & Metso, 2004). Therefore, it may be difficult to ascertain the reasons behind people’s opinions, for example why some may support or object to the decriminalising of cannabis use. Scholars should, however, endeavour to understand the reasons behind the opinions to obtain a more comprehensive image of the current situation and to anticipate future developments.

A more qualitative approach to drug attitudes has been provided by studies on drug-related reportage in popular media. A few examples of such studies, focusing on cannabis, are Acevedo (2007), Månsson (2016), and Abalo (2019, 2021). Similar studies have been conducted in Finland as well but with a focus on drugs in general (e.g., Piispa, 2001; Savonen et al., 2018; Törrönen, 2004). Those media studies describe the overall atmosphere of the societal drug debate quite well, but they often emphasise the voices of journalists, politicians, and officials, while the opinions and attitudes of ordinary citizens are given less weight. Moreover, the publicity of newspapers might restrict the discussion of the highly stigmatised topic so that some opinions may remain unsaid due to the potential disadvantages to the speaker (e.g., Hakkarainen & Metso, 2004).

One way to reach the attitudes that remain hidden in the mainstream discourses is to examine online discussions. Especially on websites where users are known by pseudonyms or act completely anonymously, citizens can express their views and arguments freely, equally, and informally. Even though online discussions have been lately utilised to examine various topics related to drug use and drug culture (e.g., Barratt, 2011; Kataja et al., 2018; Rönkä & Katainen, 2017), there have been only a couple of studies on drug attitudes in online environments (Månsson, 2014; Månsson & Ekendahl, 2013). Obviously, online discussions as data sources have certain problems as well. As the researchers usually do not know the identities or the objectives of the discussants, it remains unknown whether the user pool is somehow biased, and whether some discussants are just “trolling”. Nevertheless, studies focusing on the expressions of opinions and attitudes in online discussions can supplement the knowledge about citizens’ opinions on drugs and drug policy and the ways these issues are discussed.

This study examines discussions about the 2019 cannabis decriminalisation initiative in a popular Finnish anonymous online forum, Ylilauta. The focus of the analysis is especially on argumentation which has a major impact on how influential and convincing the messages are. Analysing argumentation may also help us understand why the discussants support or object to the initiative. The main research questions are:

1) What is the overall nature of the online discussions about decriminalising cannabis use?
2) What argumentation techniques are used to support or object to its decriminalisation?
3) How do these findings differ from those based on population surveys and media coverage?

The article starts with a brief theoretical and methodological discussion, followed by an introduction to the data and their source. The analysis section first provides an overview of the cannabis discussions in the data and then analyses the most common argumentation techniques. Finally, the results are summarised and possible conclusions discussed.

## Analytical framework

This study is based on discourse analysis, a multidisciplinary theoretical framework used in different fields of study and with different research methods. In discourse analysis, language is seen as a social practice, and meanings are not considered as given, but rather, as discursively constructed and context dependent (Angermuller et al., 2014; Johnstone, 2008; see also Fairclough, 1992). Thus, discourse analysts are interested in how social order is constructed through discursive practices. Discourse analysis is part of the social constructivist approach, which has previously been applied to cannabis discourse, for example by Månsson (2014, 2016), who has used concepts of discourse theory to analyse the construction of cannabis in online discussion and print media, and Acevedo (2007), whose study is based on post-structuralist approach. Abalo (2019, 2021) has applied critical discourse analysis, a branch of discourse analysis, to analyse the journalistic construction of renegotiation of cannabis.

The viewpoints and research methods employed in discourse analysis vary widely. Our study focuses on argumentation, which has a central role in texts that aim to have a social impact. Argumentation can be defined as a communicative act complex, in which a constellation of propositions is put forward in order to resolve a difference of opinion (van Eemeren et al., 2014). In this study, our analysis of argumentation focuses on the strategies that discussants use when they formulate propositions and thus seek to promote their viewpoints. These strategies are called argumentation techniques. Argumentation theory has previously been applied to the cannabis debate by Välimaa (2017).

Our analysis of argumentation techniques is based on the classification system provided by Perelman and Olbrechts-Tyteca (1971). They divide argumentation techniques into quasi-logical arguments (e.g., comparison and identity), arguments based on the structure of reality (e.g., causal links and argument from authority), and the relations establishing the structure of reality (e.g., argumentation by example and illustration). In addition to Perelman and Olbrechts-Tyteca’s classification system, classes of fallacies such as *ad hominem* and *straw doll* arguments (e.g., Walton, 1995) are applied as well. The argumentation techniques are presented in more detail in a later section.

A common problem when analysing online forum data is that the researcher cannot be sure whether the messages represent the “real” opinions of their writers. Discussants can intentionally provoke and “troll” other users (e.g., Hardaker, 2010), as they often do in the Ylilauta forum (see the Data section). In our analysis, we focus on the viewpoints, attitudes, meanings, and argumentation techniques constructed in the forum messages as such, instead of trying to analyse the possible intentions of the discussants (see also Lahti, 2019).

## Data

The online discussions used as data in the study were collected from Ylilauta (www.ylilauta.org), which has been among the most popular Finnish discussion fora in recent years. The discussions are from October and November 2019, when the forum counted approximately 1.3 million users and 2.0 million messages monthly (Ylilauta, 2021). The website consists of approximately 50 subfora dedicated to certain themes such as news, immigration, relationships, and music. However, by far the most popular subforum is *Satunnainen* (“Random”), where the discussions can involve practically any topic. The discussions on Ylilauta are in Finnish, with English only used in the International subforum.

Despite the large number of users, Ylilauta does not serve as a perfectly representative sample of Finnish online fora. It is often described as a Finnish equivalent of the international imageboard 4chan (e.g., Haasio, 2015; Vainikka, 2019). The discussions are characterised by their quick tempo, short and carelessly written messages, polarising and provocative style, and even trolling (see also Vainikka, 2019). The public reputation of Ylilauta is quite bad, as it is known especially for illegal activities, hate speech, and other inappropriate behaviour (Vaahensalo, 2018). The peculiar culture of Ylilauta and similar imageboards is often explained by the anonymity it provides users; a great majority of the messages are sent completely anonymously, without any username or nickname. When the messages are not connected to the identity of their author, the threshold for posting inappropriate messages is significantly lowered (Neurauter-Kassels, 2011).

The main reason for choosing Ylilauta as the data source is the exceptional openness of its discussion culture. It gives room to such persons and opinions that would otherwise be marginalised in society (Haasio, 2015; Vainikka & Harju, 2019). Illegal drugs are still a strong taboo, which limits public discussion about them. People may especially refrain from expressing views supporting a more liberal drug policy, as such views sometimes cause trouble for those who express them (e.g., Hakkarainen & Metso, 2004). Anonymity grants people an opportunity to express their views without any such fears (Barratt, 2011). Moreover, discussions on Ylilauta might also have wider impact on society, as the website reaches a large audience and is a remarkable centre for internet memes and other cultural innovations (Vainikka, 2016).

Due to the anonymity of the forum, little is known about the demographics of the users. However, based on the message contents, scholars have assumed that the user pool consists predominantly of young men (Haasio, 2015; Vainikka, 2019). This point should be taken into account especially since young men also have the most liberal attitudes towards drugs on average (Karjalainen et al., 2020).

The study data consist of 22 discussion threads collected from the “Random” subforum between 16 October and 8 November 2019, containing initial postings on the citizens’ initiative to decriminalise cannabis use. The threads were identified by manually exploring “Random” and by carrying out various Google searches. The threads include a total of 9,094 messages, with the shortest thread consisting of 46 messages and the longest 960 messages. A few threads with less than 20 messages were intentionally left out of the data. The threads are no longer available online, but copies of them can be requested from the authors of the article. To illustrate the findings of the study, the following sections provide examples of the discussions, including the original message in Finnish and our English translation.

The argumentation analysis was limited to the first 50 messages of each thread, as a comprehensive analysis of more than 9,000 messages would have been unnecessarily laborious. Two of the threads included less than 50 messages, meaning that a total of 1,092 messages were analysed. On a practical level, the analysis was conducted in three phases. First, the messages were closely read through. After that, the different argumentation strategies and techniques in the data were marked and coded using Atlas.ti software. Finally, discourse analysis and argumentation theory were used to analyse the argumentation categories on a more detailed level.

When utilising online discussion data, one should also take ethical considerations into account. As Ylilauta can be used without prior registration and reaches a large audience, we regard it as an open and public website. Consequently, the discussions can be used as research data without a consent from the website owner or users. Worth noting is also the fact that the messages are anonymous, and the identities of their senders cannot be recognised. Therefore, using messages for research purposes should cause no harm to their senders. (For online research ethics, see Franzke et al., 2020.)

## Overview of the discussions

The main topic of discussions in the data is the citizens’ initiative to decriminalise cannabis use: the collection of signatories, its future consideration in the Finnish Parliament, and the possible societal impacts of decriminalisation. The discussions are not always limited to decriminalisation, as creating legal cannabis markets and decriminalising the use of other illegal drugs are also debated in the forum. This may in part have to do with the fact that not all the forum users are aware of the exact contents of the initiative or the difference between decriminalisation and legalisation, but some discussants might have seen the initiative as a possibility to engage in a more general discussion on Finnish drug policy. Furthermore, the discussions touch on other themes related to drug attitudes and opinions, like the risks of using drugs or the societal drug problem and its treatment.

There are some differences between the contents of the threads. The earliest threads were established before 50,000 signatures, the minimum count for the Parliament to deliberate on the initiative, had been collected. These threads followed the development of the signature count and discussed the reasons to sign or refuse to sign the initiative. When the required signature count had been reached, the discussions turned to the initiative’s chance of being accepted in the Parliament. New threads were found especially when remarkable politicians gave statements on the initiative in newspaper interviews. The latest threads were increasingly about politics in Finland generally, for instance the functionality of the citizens’ initiative system and the tensions between the political parties.

The messages in the data were categorised into five groups based on their opinion of the initiative. Of the 1,092 messages, 80 (7.3%) support the initiative strongly, 240 (22.0%) support it, 454 (41.6%) take a neutral position or do not express a clear opinion, 185 (16.9%) object to the initiative, and 133 (12.2%) object to it strongly. Examples of the five categories are presented in Examples 1–5. By strongly supporting or objecting to the initiative, we mean using provocative, offensive, racist, and affective expressions (Example 1) or suggesting exceptionally radical views or actions (Example 5). The effort at measuring the strength of the wording in the messages is obviously somewhat ambiguous, and it is merely meant to give an overview of the distribution of supporting and objecting messages in the data.

1) Ei kyllä mene ymmärykseen kenen idiootin mielestä on parempi että nistien rahat menee neekereille kuin että menis valtion kirstuun veroina.
I cannot understand who the idiot prefers that junkies’ money go to niggers rather than to the government as taxes.
2) Allekirjoitettu! En polta kannabista mutta haluan että se laillistetaan.
Signed! I don’t smoke cannabis but I want it to be legalised.
3) Aivan sama mulle laillistetaanko vai ei, en ole itse kiinnostunut.
It is completely the same for me whether it will be legalised or not, I myself am not interested.
4) mitä enemmän tätä pakotatte sitä vähemmän tekee mieli allekirjottaa ☺
the more you push this [initiative], the less I feel like signing ☺
5) Nistit hirteen!
Hang the junkies!

**Figure 1.**
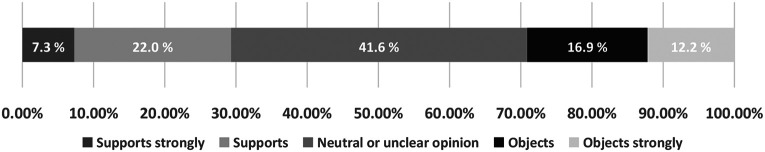
Data messages (*N* = 1,092) classified based on their opinion about decriminalising cannabis use.

Figure 1 shows that the supporters of and objectors to the decriminalisation of cannabis are almost equally numbered in the data, each group having posted approximately 29% of the messages. This means that the supporters are slightly overrepresented in the data, as 42% of the Finnish population support decriminalisation and 58% object to it (Karjalainen et al., 2020). Strong opinions are more common among the objectors (12.2%) than the supporters (7.3%). The high number of messages classified as neutral is mostly because the author’s opinion remains unclear in the message. For instance, many discussants only commented on other messages, without expressing their own opinion on the topic. Few discussants clearly positioned themselves as neutral.

In this article, we use the terms *supporter* and *objector* for the authors of the messages supporting and objecting the initiative, regardless of whether they do so in a strong manner or not. The terms are not unproblematic, as we cannot be sure about the real motivations of the authors. However, the term *objector* does not mean that the person who has written the message is necessarily against the initiative, but refers to the party that is constructed in the discussion. Similarly, the term *supporter* refers to the party of the debate in the discussion forum. Nevertheless, we have decided to use these terms to illustrate the fact that the messages in the data are quite clearly divided into two opposing sides.

## Argumentation techniques

This section presents and analyses the argumentation techniques used to support or object to decriminalisation. The most common techniques and their prevalence are assembled in Table 1. In addition to them, the data include a few techniques used less frequently. Nevertheless, it should be noted that the sum of all techniques remains relatively low with respect to the data size (1,092 messages). This can in part be explained by the numerous messages that do not express an opinion, and therefore, do not make an argument. However, plenty of messages also state opinions without providing proper reasoning.

**Table 1.** The most common argumentation techniques and their prevalence in the data (*N* = 1,092).

**Argumentation technique**	**Count**	**%**
Ad hominem	180	16.5
Straw doll	31	2.8
Comparison	58	5.3
Cause–consequence relation	30	2.7
Means–end relation	29	2.7
Argument from authority	30	2.7
Example and illustration	35	3.2

### Ad hominem

The most common argumentation technique used in the data is argumentum ad hominem. It is a fallacious strategy wherein the speaker attacks the character, motive, or some other attribute of the person making the argument instead of the argument itself (Walton, 1995). The frequency with which such attacks were made on Ylilauta is quite surprising, as the users do not know anything about each other’s personalities. Hence, the characteristics of a certain person or group presented in the ad hominem arguments are presumptions, not verified attributes. However, ad hominem arguments are commonly used in other online discussions as well (Lahti, 2019).

One strategy that appears quite often in the data involves questioning the mental capability or health of the opposing party. This was usually done through expressions like *tyhmä* (“stupid”), *tollo* (“fool”), *idiootti* (“idiot”), *matala äö* (“low IQ”), *vammainen* (“retard”), *sekopää* (“nutcase”), *autisti* (“autist”), *aivovauriopotilas* (“brain damage patient”) and *psykoosit tulilla* (“ongoing psychosis”). Interestingly, the discussants often suggested that mental incapacity is the result of excessive cannabis or alcohol use (see Example 8 later).

Supporters of decriminalisation are repeatedly referred to with the word *nisti* (“junkie”), a Finnish slang word referring to a drug (problem) user (KS s.v. nisti). In the collection of 9,094 messages, the word *nisti* appears 1,078 times. Also, the word *narkomaani* (“narcomaniac”) and its slang variants *narkkari* and *narkki* appear 155 times. Through such word choices, the supporters of decriminalisation are accused of being drug users themselves. This is obviously an oversimplification, even though decriminalisation is strongly supported among cannabis users (Hakkarainen & Karjalainen, 2017). As there are far more decriminalisation supporters than cannabis users in Finland (Karjalainen et al., 2020), decriminalisation is inevitably supported also by many people who do not use cannabis themselves. However, labelling decriminalisation supporters as drug users might serve as an efficient way to decrease their credibility, since perceptions of illegal drugs and their users have traditionally been very negative and stigmatised. Moreover, the supporters might seem overly biased if they are assumed to support decriminalisation to benefit from it themselves.

The supporters’ assumed drug use is also often connected to other negative qualities, such as uncleanliness, sickness, slow-wittedness, inefficiency, and carelessness as well as being unemployed, shunning work, and being dependent on social support. These qualities were usually expressed verbally in the data, like in Examples 6 and 7, but occasionally objectors also attached related images to their messages. A certain photo of an untidy, hollow-eyed youth, representing a stereotypical cannabis user, appears several times in the data.

6) Huutista. Tämä kansalaisaloite on varmaan isoin juttu minkä nistit ovat saaneet aikaiseksi ja mitään hyötyä siitä ei ole.
Laughing out loud. This citizens’ initiative might be the biggest thing that junkies have achieved, and yet there is no use for it.
7) Ketään ei kiinnosta muutaman työtä vieroksuvan nistin mielipide.
Nobody is interested in the opinion of a few job-avoiding junkies.

Those who object to decriminalisation are most often called as *lammas* (“sheep, lamb”), with the word appearing 130 times in the whole data of 9,094 messages, and *juntti* (“redneck, hillbilly”), which appears 80 times in the data. The word *lammas* is used metaphorically for persons lacking their own will, going along with the group (KS s.v. lammas). In the cannabis discussion, the word choice implies that any objections to decriminalising cannabis are based merely on the desire to follow majority opinion without any critical consideration of one’s own. The word *juntti* derogatorily describes a backward, conservative person unable to accept reforms (KS s.v. juntti). The word *persu*, which refers to a supporter of the nationalist-conservative Finns Party, is also occasionally used derogatorily for objectors of decriminalisation (KS s.v. persu). Furthermore, objectors are sometimes labelled as alcohol users (Example 8), which is related to the comparison of cannabis and alcohol (see the Comparison section later).

8) Sellainen matala äö juntti sieltä. Aivot selvästi jo alkoholista liuenneet.
Such a low IQ redneck there. Brain clearly already dissolved by alcohol.

Overall, however, the words used for objectors are less common and more versatile than the word *nisti* used for supporters. This might be partly because the supporters express themselves in a slightly less strong and confrontational manner on average (see Figure 1). Moreover, objectors might seem like a more heterogeneous group, making it harder to reduce them to any single term.

### Straw doll

Straw doll (or straw man) refers to a fallacious line of argumentation wherein an argument presented by the opposing side is deliberately simplified or distorted, and this modified argument is then repealed for seeming so ridiculous (Walton, 1995). The purpose of this strategy is to modify the argument so that every sensible person would object to it.

In the data, a straw doll is often an imitation of statements made by the opposing side, using prominently poor argumentation and expressions. Example 9 presents an exaggerated version of the moral panic expressed by someone opposing decriminalisation, enhanced by uppercase letters, confusing sentence structure, and a false impression of how cannabis is used (injection). Correspondingly, Example 10 makes a straw doll out of a supporter’s message using curse words, drug-user slang and a derivation of the word *öyhöttää* (to annoyingly and loudly voice one’s opinions).

**Figure 2.**
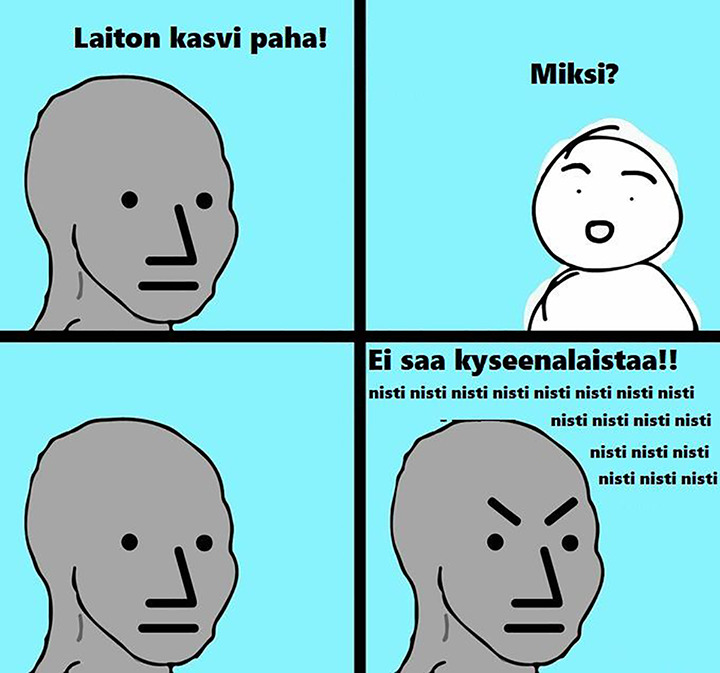
A comic strip posted as an attachment to several messages in the data. Square 1: Illegal plant evil! Square 2: Why? Square 4: Do not question!! junkie junkie junkie junkie…

9) HUUME, PSYKOOSI JA HUUME NIIN JA PSYKOOSI MUTTA PSYKOOSI MINÄ OON KYLLÄ NÄHNY KU NUORI LAPSI ON KATUOJASSA KANNABISPIIKKI KÄSIVARRESSA NIITÄ ON TUOLLA KUULE VIEROITUKSESSA NIIN!!!
DRUG, PSYCHOSIS AND DRUG, YES, AND PSYCHOSIS, BUT PSYCHOSIS, I HAVE SEEN A YOUNG KID IN THE GUTTER A CANNABIS NEEDLE IN HIS ARM, THEY ARE IN THE REHAB YES!!!
10) öyh öyh vittu miks mä en saa bleizaa gannabiz ku oon työtön vittu perkeleen persut saatana,,,,,
öyh öyh fuck, why can’t i blaze gannabiz, as i’m a jobless fuck fucking persus [Finns Party supporters] goddammit,,,,,

A few messages in the data also include a certain comic strip (Figure 2) showing an imaginary dialogue between a supporter and an objector. In the strip, the objector is represented as unable to reasonably state his opinion, repeatedly resorting to the word *nisti* (“junkie”).

Straw dolls are connected to ad hominem argumentation, as they endeavour to make not only the opposing arguments, but also the opponents themselves, look ridiculous. With such a line of argumentation, objectors are depicted as reacting irrationally and emotionally to drugs (Example 9) and being unable to provide a well-argued opinion (Figure 2), whereas supporters are depicted as unemployed cannabis users (Example 10).

### Comparison

When making comparisons, objects are evaluated through their relations to one another (Perelman & Olbrechts-Tyteca, 1971). To make effective comparisons in an argument, the choice of terms is essential (Perelman & Olbrechts-Tyteca, 1971). For example, when arguing that cannabis is harmless, it is more efficient to compare it to a more harmful substance rather than to a less harmful substance. Additionally, the identity of two objects can be used as an argument. If the objects are considered essentially identical, they should be treated equally according to the rule of justice (Perelman & Olbrechts-Tyteca, 1971).

In the data, cannabis is often compared to alcohol. This is because their legal status in Finland as well as in several other countries is remarkably different, even though many people might not see one as being significantly less healthy than the other. Similar comparisons of the two substances have been observed in previous studies on online cannabis discussions (Månsson & Ekendahl, 2013; Välimaa, 2017). Cannabis and alcohol have also been compared in scientific studies, both surveys (Hakkarainen & Metso, 2004) and medical evaluations of their adverse effects (e.g., Lachenmeier & Rehm, 2015; Nutt et al., 2007).

The comparison strategy is used especially by decriminalisation supporters in the data. According to them, cannabis is less unhealthy than alcohol and its intoxicating effect is more pleasant (Example 11). On the other hand, some objectors claim that alcohol is a better substance, invoking the same arguments (Example 12). Some objectors responded to supporters’ comparison arguments by stating that alcohol should be illegal as well, or that the harmful effects of alcohol use should be nursed by users themselves, not by society.

11) Kannabis on niin paljon terveellisempää kuin alkoholi. Et jää koukkuun, ei tule darra, mieliala paranee päiviksi käytön jälkeen. Vaikutuksen alaisena istuskelet ja naureskelet itseksesi ja syöt mässyjä.
Cannabis is so much healthier than alcohol. You don’t get addicted, do not get a hangover, the mood gets better for days after use. Under the influence, you sit and laugh by yourself and get the munchies.
12) Miks pitää sekoittaa päätä jollain kasvilla. Bissee saa kaupast vähän helpommin ja varmasti menee enemmän sekaisinkin ku jostain kasvin polttamisesta.
Why does one have to get fucked up by some plant. One gets beer more easily from the store and surely gets more fucked up than smoking some plant.

In contrast, some arguments in the data state that cannabis and alcohol are essentially identical in their effects, and thus, they should be treated similarly. The comparison made in Example 13 aims to prove that, just as legalising alcohol reduced criminality, a similar development could be expected after decriminalising cannabis. Example 14 presents an argument that the adverse effects of using cannabis cannot justify keeping it illegal, since alcohol is legal despite having similar effects.

13) Samalla tavalla viinan rikollinen järjestäytynyt trokaaminen väheni kun alkoholi taas laillistettiin (niin Suomessa kuin Yhdysvalloissa).
Similarly, organised criminality related to liquor bootlegging decreased when alcohol was legalised again (both in Finland and in the United States).
14) ihan samalla lailla alkoholikin tuhoaa ihmissuhteita, vie työpaikkoja ja syrjäyttää ihmisiä vaikka onkin laillista.
alcohol also destroys relationships, takes jobs and displaces people, though it is legal.

Even though comparing cannabis and alcohol is seemingly justified, it is often based on unjustified premises. Some discussants presume that all people have a need to use some intoxicant, and therefore, they label objectors as alcohol users. Supporters, in contrast, are sometimes told to use alcohol instead of cannabis, implying that the substances are related alternatives. However, Finnish studies suggest that cannabis users actually consume more alcohol than average citizens (Hakkarainen & Karjalainen, 2017).

Occasionally, the discussants compared the effects of cannabis to those of other illegal drugs as well as to legal psychoactive substances, such as tobacco, snuff, coffee, or sugar. Moreover, some compared cannabis to issues previously illegal but that are legal nowadays, for example voting rights for women, abortion, and homosexual marriages. These comparisons aim to demonstrate that the illegal status itself should not be used as an argument for keeping it illegal.

### Causal links: cause–consequence and means–end relations

Objectors of decriminalisation use cause–consequence relations in their argumentation, while supporters use means–end relations. Both relations are based on causal links, but means–end relations include intentionality, and thus, present certain actions as a means to achieve desired ends (Perelman & Olbrechts-Tyteca, 1971). The real effects can then even be the opposite of the desired effects.

Objectors in the data typically assume that decriminalisation increases cannabis use (Example 15) and its negative effects for users and society, for example through increasing the number of health issues (Example 16) and criminality or decreasing employment and productivity. They likewise assume that using cannabis will lead to using other drugs, referencing the gateway drug effect argument. Furthermore, they believe that decriminalising cannabis will lead to decriminalising and legalising other drugs as well. Supporters, on the other hand, present decriminalisation and legalisation as a means of achieving desired ends, like more effectively helping drug addicts (Example 17), decreasing criminality, increasing tax revenues for the government, and regulating the quality of cannabis products. These arguments do not claim that cannabis use is harmless or beneficial but seek to reduce its harms.

15) Se on fakta, että dekriminalisointi lisää huumeidenkäyttöä ja ongelmia niistä.
It is a fact that decriminalisation increases drug use and the problems caused by them.
16) Ei koskaan vaarallisia huumeita laillisiksi. Olen lukenut liian monta juttua psykoosesista jo ensimmäisellä käyttö kerralla ja sitten on loppu elämä pilalla.
Never legalise dangerous drugs. I have read too many stories of psychoses already when trying them for the first time, and then the rest of your life is ruined.
17) Suurena ongelmana tällä hetkellä on ongelmakäyttäjien saaminen asialliseen hoitoon. Pelkästään käytön dekriminalisointi (eli rikoksen poistaminen) laskisi addiktoituneiden kynnystä hakea hoitoa. Samalla käyttö saataisiin sosiaalisesti hyväksyttävämmäksi, jolloin ongelmakäyttäjät uskaltautuisivat tulla esiin ongelmiensa kanssa.
Currently, a major problem is to get problem users properly treated. Merely decriminalising the use (i.e., removing the crime) would make it easier for addicts to seek treatment. Simultaneously, using [drugs] would be socially more acceptable and problem users would be encouraged to come out with their problems.

### Argument from authority

The argument from authority uses the acts or opinions of a person, group of persons, an institution, or public opinion as the means to support a claim (Perelman & Olbrechts-Tyteca, 1971). This kind of argumentation can be fallacious if the authority being appealed to is not relevant for the topic at hand, for example citing an incorrect field of expertise (Walton, 1995).

In the data, the discussants often appealed to such authorities as the World Health Organization, United Nations, Finnish Institute of Health and Welfare, and universities, which can be regarded as relevant and trustworthy authorities concerning the topic. Politicians and other distinguished members of society are also mentioned as authorities. In Example 18, the writer cites the statement of Jussi Halla-aho, leader of the Finns Party (called Mestari “Master” by some of his most enthusiastic fans). This kind of argumentation could be regarded as fallacious; even though politicians serve as general societal authorities, they do not necessarily have expertise on the particular topic of discussion.

18) Mestarin sanoja lainaten, Me emme tarvitse Suomeen alkoholin lisäksi mitään muuta päihdeongelmaa.
Quoting Mestari, We do not need any other intoxicant problem in Finland in addition to alcohol.
19) Onneksi on faktoja ja tilastoja siitä, että kannabis on vaarallinen päihde.
Fortunately, there are facts and statistics showing that cannabis is a dangerous intoxicant.

References to the authorities are often imprecise. The authors appeal to public opinion or talk about facts and statistics (Example 19) without specifying the source of the information. This inadequate referencing resembles the “weak science discourse” found in Swedish news media reportage by Abalo (2021). It is typical not only of the cannabis discussion but of online discussions in general (Lahti, 2019).

### Examples and illustrations

Argumentation by example means that examples are used to establish a rule or make a generalisation (Perelman & Olbrechts-Tyteca, 1971). Thus, the contents of the example are not regarded as unique but as the manifestation of a certain rule. Whereas the role of an example is to establish a rule, the role of illustration is to strengthen and illustrate a rule already presented (Perelman & Olbrechts-Tyteca, 1971).

Supporters of decriminalisation use other countries, where the effects of amendments have been positive, as examples and illustrations. Portugal, which decriminalised the use of all drugs in the early 2000s, is provided as an example of the fact that drug use does not necessarily increase and that drug-related deaths might decrease significantly. Canada, Uruguay, and certain states in the USA that have legalised cannabis markets are used to illustrate the positive effects on the economy and the quality of cannabis products.

Discussants also use their own experiences or observations as examples. Objectors highlight the negative effects of cannabis use experienced by the writers themselves or by their friends and relatives; this strategy is also often used in print media (Månsson, 2016). Supporters of decriminalisation justify their argument by saying they have experienced no harmful effects as a result of using it (Example 20). The possible enjoyable or other positive effects of cannabis, however, are rarely used as an argument (cf. Abalo, 2021; Engel et al., 2020).

20) – – Itse olen töissäkäyvä insinööriukko ja toisinaan polttelen pajaria, kun vaihto-opiskellessa jäi ‘tapa’. Ei vaikuttanut valmistumiseen, työnsaamiseen tai työssäkäyntiin. Elelen vallan kunnollista ja lainkuuliaista elämää, mutta joudun olemaan rikollisten kanssa tekemisissä, kun ostan tuotetta.
– – I myself am a working engineer man and smoke pot occasionally because I acquired the “habit” while being an exchange student. It has not affected my graduation, employment or working life. I live quite a respectable and law-abiding life, but I have to be involved with criminals when buying the product.

## Conclusion

The cannabis discussions on Ylilauta are quite highly polarised between the supporters of and the objectors to decriminalisation. The parties often seemingly aim to insult and provoke each other with strong rhetoric rather than trying to convert the opposite side through valid argumentation. Occasionally, the messages even resort to expressions that can be considered hate speech.

Argumentation in the discussions is often insufficient. A notable number of claims in the messages are not reasoned in any solid manner, and the most frequent argumentation technique in the data is the fallacious ad hominem. Some users provide sufficient reasoning to properly support their views, referring to scientific studies and appealing to authorities with expertise, even though the references to them are often imprecise.

The supporters and the objectors use somewhat similar argumentation structures, though in opposite ways. The supporters are labelled drug users by people calling them junkies, whereas the objectors are called weak-willed lambs or backward rednecks. The objectors justify their arguments through cause–consequence relations, claiming that decriminalisation will increase cannabis use and its adverse effects, while the supporters justify their arguments through means–end relations, describing decriminalisation as a way to support problem users. Examples and authorities are cited to support both views.

How do these findings supplement previous knowledge about drug opinions and drug policy debate, based on population surveys and media coverage studies? First, the topic seems to arouse very powerful emotions, which are usually kept hidden in the public debates but can be expressed openly in anonymous online discussions. Second, fallacious argumentation techniques such as ad hominem and straw doll are obviously avoided in public discourses. Their commonness in online discussions might indicate that a remarkable percentage of citizens ground their opinions on feelings rather than rational and analytical thinking. This remains unnoticed in population surveys, where reasons or arguments for the opinions are not asked.

Worth noticing is also that cannabis is compared to and contrasted with alcohol in Ylilauta discussions, similarly to a popular Swedish forum (Månsson & Ekendahl, 2013). This might indicate that participants in the fora view alcohol as a more relevant parallel for cannabis than other illegal drugs. This would be in line with population surveys, which have found that a growing number of Finns view cannabis differently than other drugs in terms of both its risks and the punishment for using it (Hakkarainen & Karjalainen, 2017; Karjalainen et al., 2020). However, one should note that such comparisons are made especially by those who support decriminalisation.

Polarisation, strong rhetoric, and insufficient argumentation are common problems associated with many kinds of online discussions (Lahti, 2019), but the topic undoubtedly has an influence as well. As drugs have long been stigmatised or even demonised in society (e.g., Christie & Bruun, 1985), discussing them might provoke strong emotional reactions. The lack of sufficient argumentation might also be the result of limited knowledge about the topic. Many citizens receive their information about drugs mostly from the news and drug education in schools, where the most negative aspects of drugs, such as problem use and drug-related criminality, are emphasised (Hakkarainen et al., 2015). Giving more visibility to users who experience little harm from cannabis use, as well as the reasons for using cannabis despite the potential harms, could diversify the public image of cannabis and destigmatise the subject, allowing for a less emotional and more rational debate on its legal status (see also Abalo, 2019; Engel et al., 2020).

The results of this study should not be generalised to all online discussions about cannabis. Ylilauta has a peculiar culture, characterised by informal and carefree attitudes and a lack of political correctness, which often leads to more polarised debates than on other websites. However, the anonymity on the website provides discussants with a chance to express their opinions more freely than in public discourses, where particularly the voices demanding a more liberal drug policy might be silenced (Barratt, 2011; Hakkarainen & Metso, 2004). Therefore, the messages might express the opinions of their authors even more genuinely than the statements presented in public debates.

Nevertheless, online discussions about drug policy should also be studied elsewhere on the internet and in social media. Qualitative analyses of online discussions could supplement the general knowledge of citizens’ drug opinions and especially the reasons behind them. A comprehensive understanding of drug opinions is important when assessing the current drug policy. The question of the legal status of drugs is not simple, as it brings into conflict such crucial values as freedom, health, and security. However, since cannabis use is increasing rapidly both in Finland and in many other countries, the question concerns a growing number of citizens. Therefore, it is vital to be able to discuss the topic as openly, neutrally, and rationally as possible.
